# Ileal Tubulo-Villous Adenoma Causing Small Bowel Obstruction in a Virgin Abdomen

**DOI:** 10.7759/cureus.17421

**Published:** 2021-08-24

**Authors:** Obteene Azimi-Ghomi, Gerardo Kahane

**Affiliations:** 1 General Surgery, Kendall Regional Medical Center, Miami, USA; 2 General Surgery, Aventura Hospital and Medical Center, Aventura, USA

**Keywords:** small bowel adenoma, ileum, ileal adenoma, small bowel obstruction, virgin abdomen, sbo, sbo-va

## Abstract

Small bowel obstructions (SBO) are a common surgical problem accounting for up half of all emergency laparotomies in the United States. SBO in the virgin abdomen (SBO-VA) presents surgeons with a unique predicament as historical teaching has mandated operative exploration in these situations due to their association with more sinister etiologies. More recent research has demonstrated that this may not be the case, with adhesive disease comprising the majority of SBO-VA. Small bowel neoplasms however comprise a considerable portion of SBO-VA. Small bowel tumors comprise around 0.5%-2% of all gastrointestinal tumors, with adenomas being the most common type of benign small bowel tumor. These lesions are most commonly encountered in the duodenum, typically involving the peri-ampullary region, Their incidence decreases with descent down the gastrointestinal tract, and are least commonly found in the ileum. Ileal adenomas have been increasingly described in the literature with the rise of advanced imaging and endoscopic capabilities. The vast majority of these lesions remain asymptomatic; however, they have been reported to undergo malignant transformation resulting in obstruction and intussusception. Small bowel obstruction due to ileal adenomas in the absence of malignancy is exceedingly rare, with only one previously reported case in the literature. We describe a case of an SBO-VA secondary to ileal stricture caused by a tubulo-villous adenoma. We then discuss the topics of SBO and SBO-VA, specifically regarding their etiology and historical and modern management, with a particular focus on the diagnosis and management of small bowel neoplasms, specifically small bowel adenomas.

## Introduction

Small bowel obstructions (SBO) are a commonly encountered surgical problem resulting in thousands of hospital admissions and resultant operative interventions yearly in the United States. SBOs are most commonly due to adhesive small bowel disease in patients with a previous history of abdominal operations, with up to 70% of these cases resolving with conservative management. Historically, presentation of SBO-VA mandated surgical exploration due to the concern for neoplastic obstruction and the belief that adhesive disease was not present. Recent reviews now demonstrate that adhesive small bowel disease is quite prevalent in SBO-VA. Small bowel neoplasms however comprise a considerable percentage of SBO-VA. We discuss a case of an SBO-VA secondary to an ileal tubulo-villous adenoma that developed a stricture. Following a discussion of the workup and operative management of our case, we discuss an overview of small bowel obstructions, particularly regarding the management of SBO-VA, with an attention towards small bowel adenomas and adenocarcinomas.

## Case presentation

A 64-year-old male with a past medical history of hypertension presents to our facility with complaints of worsening abdominal pain for the last week. The patient reported that he had been experiencing abdominal discomfort that he initially attributed to gastric reflux, for which he was prescribed anti-reflux medications by his primary care physician, without improvement of symptoms. He stated that the abdominal pain was initially intermittent but subsequently progressed to persistent and constant abdominal pain. He also reported associated abdominal distension during the past week, with nausea and vomiting that began one day prior to presentation. The patient additionally reported not having a bowel movement for the past three weeks. The patient denies previous abdominal operations as well as ever undergoing a colonoscopy or upper endoscopy. He denies any family history of cancer. He reported denied a history of tobacco, alcohol or illicit substance use. He reported his occupation as a mechanic.

The patient’s vitals were stable on first encounter, apart from mild tachycardia of 103 beats per minute that normalized with intravenous crystalloid resuscitation. A complete blood count as well as chemistry panel were within normal limits. Lactic acid level was 1.2 mmol/Liter. 

The patient’s physical exam was significant for abdominal distension that was tympanic to percussion in all four quadrants. The patient’s abdomen was diffusely tender to palpation, with increased tenderness in the epigastrium, but without peritonitis. No hernia was palpated in the bilateral inguinal regions. There were no prior surgical scars on the abdomen or pelvis. 

**Figure 1:**
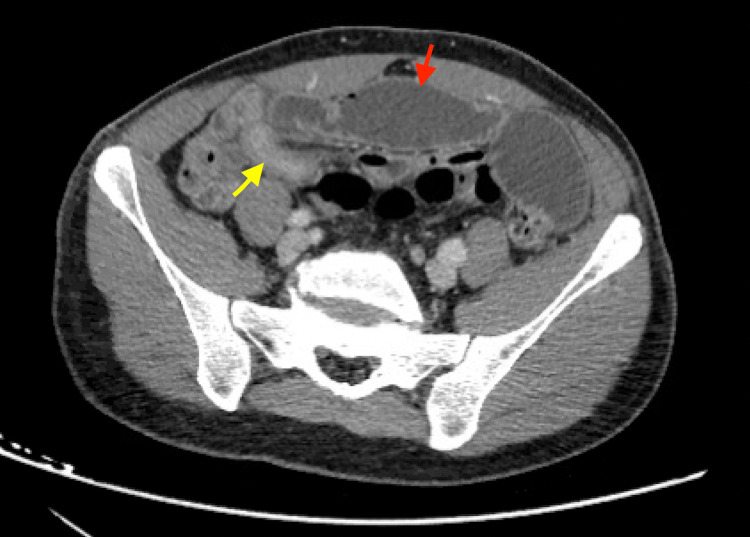
CT abdomen and pelvis demonstrating the thickened segment of ileum (yellow arrow) resulting in the transition point located in the right lower quadrant, with dilation of proximal loops of small bowel (red arrow).

Computed tomography (CT) of the abdomen and pelvis with intravenous contrast only demonstrated a high-grade obstruction with dilated, fluid-filled loops of small bowel and a transition point in the right lower quadrant (Figure 1). The transition point demonstrated a segment of circumferential wall thickening without fat stranding (Figure 1). There was no free air or fluid noted. 

**Figure 2:**
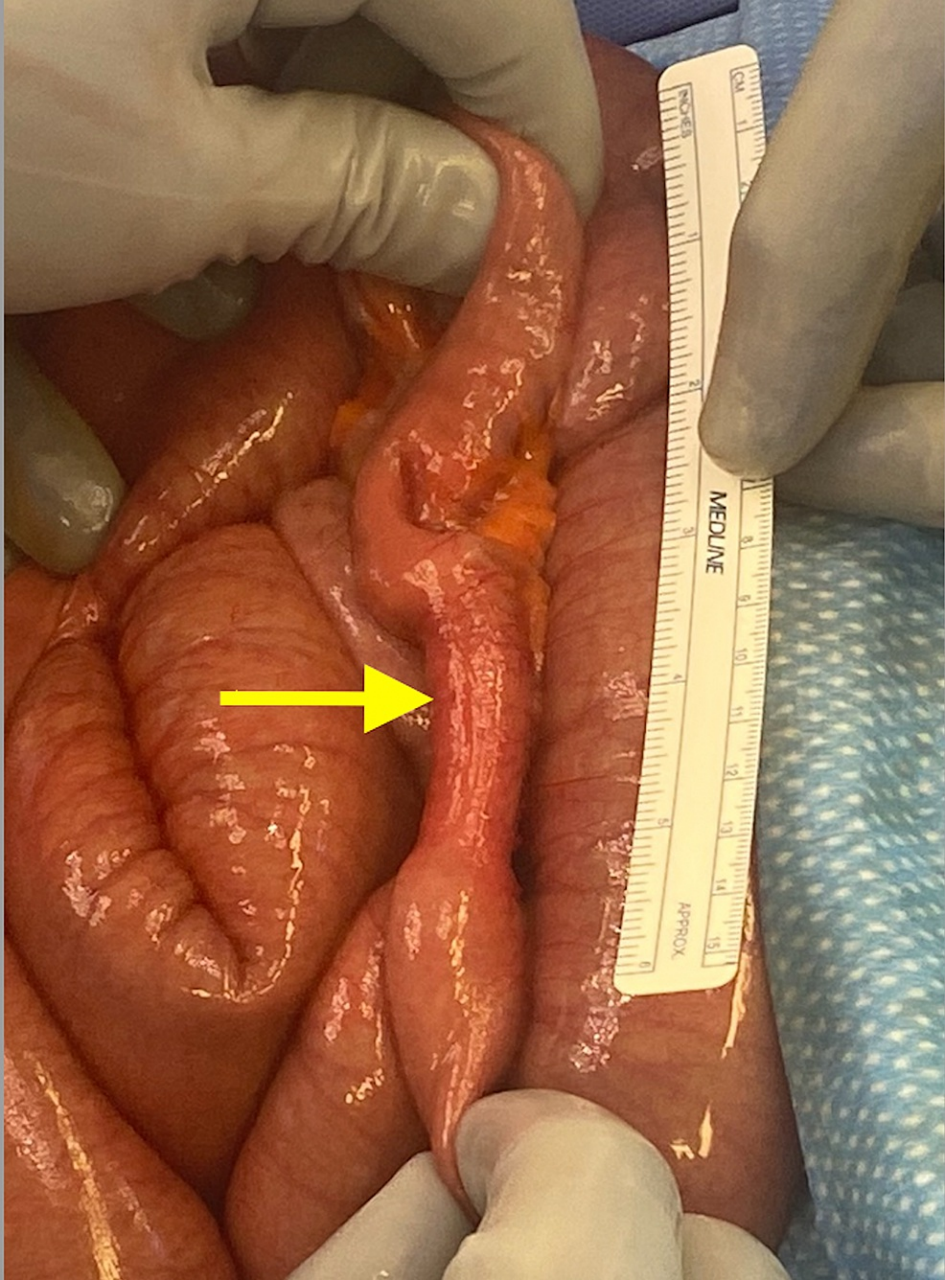
8 cm segment of ileum with obstructive stricture (yellow arrow).

**Figure 3:**
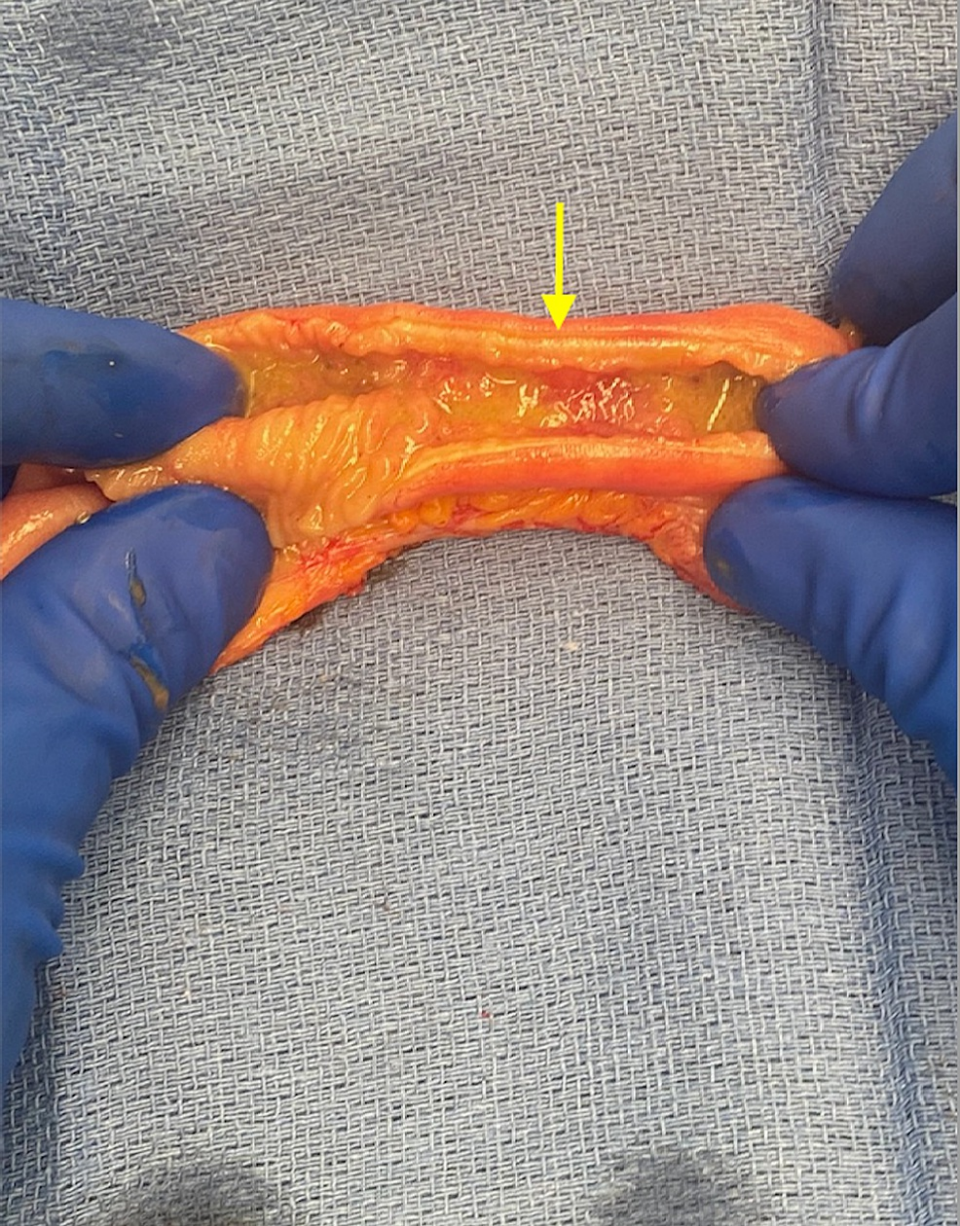
Dissection and inspection of the strictured segment of ileum, demonstrating mural thickening, predominantly in the mucosa and submucosa, with fibrosis of the wall (yellow arrow). Mucosal erythema and ulceration are also noted in the strictured segment (arrow).

**Figure 4:**
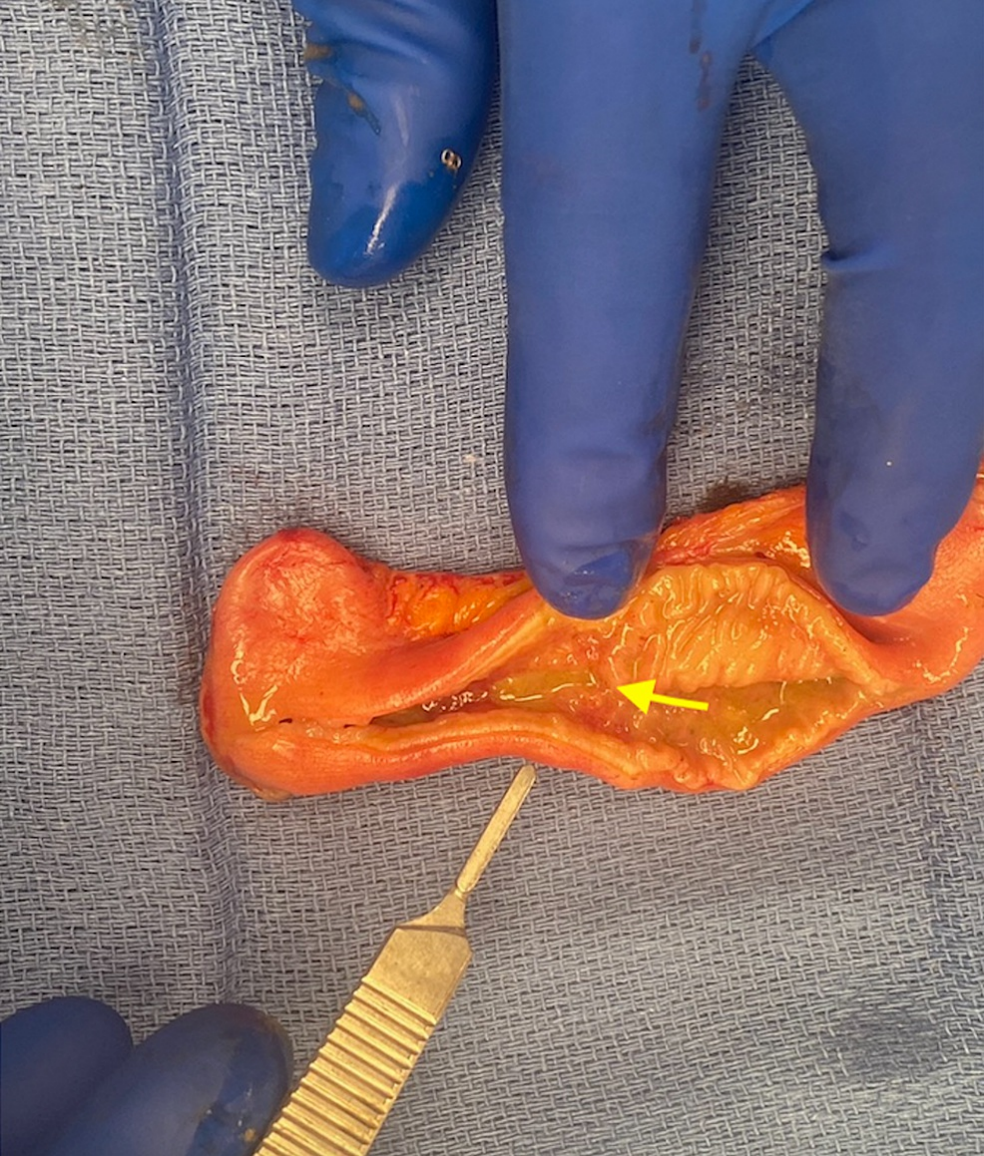
Inspection of the strictured segment of ileum, demonstrating mural thickening, predominantly in the mucosa and submucosa, with fibrosis of the wall (yellow arrow). There is normal mucosa and gross mural anatomy adjacent to the strictured segment of ileum.

The patient underwent an exploratory laparotomy where an 8 cm stenotic segment of proximal ileum was noted with decompressed small intestine distally (Figure 2). The stenotic segment was palpated and noted to be hard and fibrotic. No intestinal contents were able to be milked through this stricture distally. We decided to resect the stenotic segment and perform a primary anastomosis, which was performed without issue using staples. The remaining small bowel as well as the large bowel were inspected, and no further lesions were noted. The abdomen was closed in a standard fashion. On the backtable, the resected segment of small bowel was inspected and a thick fibrotic submucosal layer was noted with mucosal ulceration (Figures 3, 4). 

The patient’s post-operative course was rather unremarkable. The patient was ambulating on post-operative day #1, with bowel function returning on post-operative day #3. The patient was started on a liquid diet, which he tolerated well and was advanced accordingly. The was subsequently discharged on post-operative day #5. Histopathology of the resected segment of bowel demonstrated circumferential tubulo-villous adenoma with ulceration and fibrosis. No foci of carcinoma were noted.

## Discussion

Acute SBO is a common surgical problem, accounting for 15% of surgical admissions for abdominal pain in the United States, and accounting for almost half of all emergency laparotomies [1-4]. Approximately 80% of patients with SBO have undergone previous intra-abdominal operation [5]. Intra-abdominal adhesions are the most common cause of SBO, seen in 60%-75% of cases [5,6]. Other less common causes include hernias, malignancy, inflammatory bowel disease, foreign bodies, and volvulus [6,7]. Approximately 70% of small bowel obstructions due to adhesions are successfully managed non-operatively [6,7]. Surgical intervention is performed in SBOs that fail to resolve with non-operative management, as well as for SBOs presenting with signs and symptoms of bowel ischemia or perforation, which is seen in up to 40% of cases [6,7]. 

Small bowel obstruction in patients with a virgin abdomen (SBO-VA) have historically been operative situations due to the belief that more sinister etiologies are behind the obstruction [8,9]. Compared to the roughly 30% operative rate for adhesive SBO, SBO-VA have a reported operative rate of between 40-100% [8,9]. Recent systematic reviews have demonstrated mandatory operation may no longer be the case [10-14]. These reviews demonstrated that the etiology of SBO-VA was comparable to SBO seen in patients with previously abdominal procedures [10-14]. A systematic review by the WSES released in 2021 recommended that SBO-VA may be treated using existing management guidelines for adhesive SBO [9]. It should be noted however, that SBO-VA carry a relatively high association with neoplastic etiologies, with 4-41% cases caused by neoplastic lesions [10-14]. 

Small bowel tumors are rare, accounting for <3% of all gastrointestinal tumors in the United States [15]. Benign tumors comprise 0.5-2% of all gastrointestinal (GI) tumors, consisting mainly of adenomas, lipomas, hamartomas, benign gastrointestinal stromal tumors (GIST), and leiomyomas [15]. Small bowel adenomas and adenocarcinomas are typically present in patients 50 years and older. Younger age involvement is most commonly seen in those afflicted with congenital polyposis syndromes [15,16]. Adenomas are the most common type of benign small bowel tumor, and are categorized as tubular, tubulo-villous, villous or Brunner’s gland-associated [15-17]. These adenomas, like their counterparts seen in the colon and rectum, have a potential for malignant transformation. The location of small bowel adenomas mirrors that of small bowel adenocarcinoma, with the duodenum being the most common location, particularly in the peri-ampullary region [15,16]. These adenomas are typically villous in etiology [15,16]. With progression distally, adenomas are found with decreasing incidence, and are least commonly found in the ileum, where they are usually tubular in etiology [16]. Adenomas are most commonly solitary and <2 cm in diameter [15-17]. These lesions are typically sessile. Villous adenomas are commonly larger than tubular adenomas, reaching sizes of > 3 cm [15-17]. They tend to be broad-based lesions with a cauliflower appearance on both imaging and gross examination [15-17]. Small bowel adenomas are commonly asymptomatic, being identified incidentally on imaging, or during surgery or pathological examination of resected small bowel for other pathologies [15-17]. When symptomatic, they typically present with obstruction, though this is usually rare in the absence of carcinoma, as lesions large enough to cause an obstructive physiology have concomitant carcinoma [15-17].

The presence of multiple adenomas is uncommon, but when present typically associated with familial polyposis conditions such as Familial Adenomatous Polyposis (FAP), Turcot Syndrome, Gardner Syndrome, Peutz-Jeghers Syndrome (PJS), and Cronkhite-Canada Syndrome (CCS) [15-17]. Multiple adenomas tend to occupy a single bowel segment [15-17]. These lesions can vary in size and may be both sessile and pedunculated [15-17].

Small bowel lesions were historically identified using upper gastrointestinal fluoroscopic studies with small-bowel follow-through (SBFT) [16-18]. SBFT however has demonstrated a low yield. Enteroclysis is the superior fluoroscopic study for detecting small lesions [16-18]. Computed Tomography (CT) of the abdomen and pelvis with oral and intravenous contrast has now replaced fluoroscopic studies due to the former’s ease of administration. Depending on size and location, small bowel adenomas may either be undetected, or appear as a smoothly outlined intraluminal filling defect or mass on contrast radiography or CT [17]. Larger lesions, typically villous adenomas, may present as broad-based cauliflower-like filling defects in imaging [15,17]. Suspected small bowel lesions on CT imaging should be further assessed with CT-enterography (CTE) or magnetic resonance enterography (MRE), which are the preferred imaging studies for small bowel tumors [15,17,18]. Small bowel tumors may exhibit distinctive signal intensity characteristics that differ from those seen in the adjacent small bowel wall, improving tumor conspicuity [17].

Asymptomatic polyps smaller than 2 cm and those not associated with genetic polyposis syndromes can be observed unless they are amenable to endoscopic intervention [15-18]. Adenomatous polyps > 2-3 cm have a higher potential for obstructive symptomatology and malignant conversion [17-19]. Therefore, surgical or endoscopic resection is recommended [18]. When adenocarcinoma is highly suspected or diagnosed during surgical intervention, segmental resection with wide local excision of the associated mesentery is recommended, in order to sample the draining nodal basin [18,19]. There is no consensus on margin size, but usually, at least 5cm margins are recommended [20]. Depending on staging, adjuvant chemotherapy may be required [20].

Our case report is unique in that there have very rarely been described incidences of ileal adenomas resulting in small bowel obstruction. There have been several cases of ileal adenomas resulting in ileo-cecal or ileo-ileal intussusception [21,22]. However, there has been only one previous case report of an ileal adenoma resulting in stricture formation and small bowel obstruction, similar to our case [23]. The patient in this case underwent an EGD and colonoscopy, which were negative. The patient’s obstructive lesion was diagnosed with SBFT, and surgical exploration demonstrated a 4 cm tubulo-villous adenoma resulting in stricture formation [23].

## Conclusions

Small bowel obstructions are an extremely common surgical dilemma. Historically, the management of SBO secondary to adhesive small bowel disease and SBO-VA have been vastly different, with concern for obstructive tumors mandating operative exploration in SBO-VA, especially those without hernia. This paradigm has come into question recently, with recent research demonstrating a relatively comparable percentage of adhesive small bowel disease in both SBO-VA and SBO in the previously operated abdomen. Small bowel neoplasms, however, comprise a considerable percentage of causes of SBO-VA, and should be high on the differential, especially in elderly populations and those with suspicious findings on CT and SBFT. When there is suspicion for small bowel neoplasms, delineation of these lesions can be obtained using advanced imaging modalities including computed tomography enterography (CTE) and magnetic resonance enterography (MRE).

Small bowel adenoma and adenocarcinoma are rare gastrointestinal tumors. The majority of small bowel adenomas remain asymptomatic, though these lesions carry a risk for malignant transformation as well as intussusception or obstruction. These lesions are most commonly found in the duodenum, with decrease in incidence with the further distal the small bowel goes. Ileal adenomas and adenocarcinomas are the rarest forms of small bowel adenocarcinoma. Ileal adenomas are increasingly rare and the majority are asymptomatic. When symptomatic, these lesions most commonly present with intussusception due to their close proximity with the ileocecal valve and cecum. Obstructive pathophysiology is extremely rare in ileal adenomas in the absence of adenocarcinoma, with only one other case reported in the literature. When suspected, these lesions should be managed similarly to small bowel adenocarcinoma. Operative intervention is mandated in all symptomatic lesions, as well as asymptomatic lesions >2-3 cm, tumors associated with polyposis syndromes, tumors with features suspicious for adenocarcinoma, and lesions that are amenable to endoscopic intervention.
